# Supplementary material for: Toxicological Screening of Four Bioactive Citroflavonoids: In Vitro, In Vivo, and In Silico Approaches
Source: Molecules. 2020 Dec 16;25(24):5959. doi: 10.3390/molecules25245959 (PMC7766697; doi:10.3390/molecules25245959)
Supplement: Supplementary file 1 [file molecules-25-05959-s001.pdf]

SUPPLEMENTARY INFORMATION

Tabla S1. Hematic cytometry values and coagulation time of animals treated with QRT

Parameter	Control	Dose of QRT	
		50 mg/kg	300 mg/kg
Red series			
Erythrocytes (10^6/μL)	7.47±0.45	3.65±0.60*	8.12±0.32
Hb (g/dL)	14.05±0.25	14.55±0.65	14.67±0.45
Hto (%)	41.10±1.10	45.50±2.30	45.73±1.15
MCV (fL)	55.20±1.70	56.45±3.05	56.37±0.90
MCH (pg)	19.60±0.75	18.05±0.85	18.07±0.20
MCHC (g/dL)	34.20±0.30	32.00±0.20*	32.07±0.20*
RBCDW (%)	14.50±1.00	17.95±0.45	16.80±0.20
White series (10^3/μL)			
Leukocytes	2.54±0.05	3.65±0.60	4.21±0.38
Neutrophils	25.00±4.00	31.00±3.00	23.66±8.50
Lymphocytes	71.50±2.50	68.50±2.50	74.67±8.50
Monocytes	1.00±1.00	0.50±0.50	1.33±0.00
Eosinophils	2.00±1.00	0.00±0.00	0.33±0.00
Basophils	0.00±0.00	0.00±0.00	0.00±0.00
Hemostasis			
Platelets (10^5/μL)	831.00±225.00	759.00±22.00	665.00±125.33
PT (sec.)	22.10±0.40	16.20±2.00	16.00±2.65
APTT (sec.)	17.15±0.35	14.55±0.25	15.75±1.45

Values are presented as mean± standard error. n=3, *$p<0.05$ with respect to vehicle. Hb: hemoglobin, Hto: hematocrit, MCV: mean corpuscular volume, MCH: mean corpuscular hemoglobin, MCHC: mean concentration of corpuscular hemoglobin, RBCDW: red blood cell distribution width, PT: prothrombin time and APTT: activated partial thromboplastin time.

Tabla S2. Hematic cytometry values and coagulation time of animals treated with HESP

Parameter	Control	Dose of HESP	
		300 mg/kg	2000 mg/kg
Red series			
Erythrocytes ($10^6/\mu L$)	7.72±0.26	7.90±0.30	8.04±0.22
Hb (g/dL)	14.03±0.26	14.20±0.37	14.20±0.36
Hto (%)	44.13±0.60	44.96±0.91	43.26±0.89
MCV (fL)	57.36±1.99	57.06±1.87	53.86±0.37
MCH (pg)	18.20±0.37	18.00±0.24	17.70±0.24
MCHC (g/dL)	31.80±0.48	31.56±0.64	32.83±0.35
RBCDW (%)	17.63±0.85	17.00±0.61	17.83±1.50
White series ($10^3/\mu L$)			
Leukocytes	2.60±0.62	4.50±0.89	6.68±0.72 *
Neutrophils	20.87±2.13	0.97±0.79 *	6.53±2.67 *
Lymphocytes	70.87±2.45	72.27±4.36	77.33±3.24
Monocytes	4.67±1.32	7.07±1.50	7.13±1.15
Eosinophils	3.60±0.29	3.60±0.76	3.67±0.54
Basophils	0.00±0.00	2.13±1.74	0.00±0.00
Hemostasis			
Platelets ($10^5/\mu L$)	681.33±12.76	720.00±125.23	628.66±28.31
PT (sec.)	17.33±0.87	17.86±0.40	17.73±0.50
APTT (sec.)	26.40±3.72	28.53±4.31	32.53±3.93

Values are presented as mean± standard error. n=3, *p<0.05 with respect to vehicle. Hb: hemoglobin, Hto: hematocrit, MCV: mean corpuscular volume, MCH: mean corpuscular hemoglobin, MCHC: mean concentration of corpuscular hemoglobin, RBCDW: red blood cell distribution width, PT: prothrombin time and APTT: activated partial thromboplastin time.

Tabla S3. Hematic cytometry values and coagulation time of animals treated with NARGE

Parameter	Control	Dose of NARGE	
		300 mg/kg	2000 mg/kg
Red series			
Erythrocytes ($10^6/\mu L$)	7.72±0.26	6.98±1.18	8.03±0.29
Hb (g/dL)	14.03±0.26	12.00±1.81	14.23±0.22
Hto (%)	44.13±0.60	36.76±5.31	44.63±0.44
MCV (fL)	57.36±1.99	53.46±1.87	57.85±1.59
MCH (pg)	18.20±0.37	17.40±0.39	18.05±0.39
MCHC (g/dL)	31.80±0.48	32.53±0.49	31.25±0.18
RBCDW (%)	17.63±0.85	17.80±1.89	18.93±0.14
White series ($10^3/\mu L$)			
Leukocytes	2.60±0.62	5.31±0.74	5.57±1.20
Neutrophils	20.87±2.13	12.93±2.05	12.67±1.12
Lymphocytes	70.87±2.45	76.10±3.41	75.77±1.38
Monocytes	4.67±1.32	8.07±0.28	7.73±0.76
Eosinophils	3.60±0.29	2.80±1.19	3.77±0.46
Basophils	0.00±0.00	0.10±0.08	0.07±0.05
Hemostasis			
Platelets ($10^5/\mu L$)	681.33±12.76	726.00±12.26	832.33±37.48 *
PT (sec.)	17.33±0.87	ND	15.46±0.71
APTT (sec.)	26.40±3.72	ND	26.26±1.85

Values are presented as mean± standard error. n=3, *p<0.05 with respect to vehicle. Hb: hemoglobin, Hto: hematocrit, MCV: mean corpuscular volume, MCH: mean corpuscular hemoglobin, MCHC: mean concentration of corpuscular hemoglobin, RBCDW: red blood cell distribution width, PT: prothrombin time and APTT: activated partial thromboplastin time.

Tabla S4. Hematic cytometry values and coagulation time of animals treated with NAR

Parameter	Control	Dose of NAR	
		300 mg/kg	2000 mg/kg
Red series			
Erythrocytes ($10^6/\mu L$)	7.72±0.26	7.79±0.27	7.41±0.11
Hb (g/dL)	14.03±0.26	14.03±0.38	13.67±0.16
Hto (%)	44.13±0.60	40.63±1.03	40.66±0.39
MCV (fL)	57.36±1.99	54.40±1.92	54.86±0.38
MCH (pg)	18.20±0.37	18.03±0.16	18.43±0.05
MCHC (g/dL)	31.80±0.48	34.53±0.10 *	33.50±0.14
RBCDW (%)	17.63±0.85	16.16±0.58	15.80±0.12
White series ($10^3/\mu L$)			
Leukocytes	2.60±0.62	2.65±0.30	4.45±0.98
Neutrophils	20.87±2.13	12.50±3.85	16.23±4.29
Lymphocytes	70.87±2.45	74.36±3.29	79.10±4.55
Monocytes	4.67±1.32	6.00±1.91	3.33±0.49
Eosinophils	3.60±0.29	3.03±0.52	1.33±0.49
Basophils	0.00±0.00	4.10±3.34	0.00±0.00
Hemostasis			
Platelets ($10^5/\mu L$)	681.33±12.76	726.00±12.26	553.00±239.61
PT (sec.)	17.33±0.87	ND	ND
APTT (sec)	26.40±3.72	ND	ND

Values are presented as mean± standard error. n=3, *p<0.05 with respect to vehicle. Hb: hemoglobin, Hto: hematocrit, MCV: mean corpuscular volume, MCH: mean corpuscular hemoglobin, MCHC: mean concentration of corpuscular hemoglobin, RBCDW: red blood cell distribution width, PT: prothrombin time and APTT: activated partial thromboplastin time.
